# Efficacy of Lipid Ratios and Platelet Distribution Width for Assessment of Liver Fibrosis in Patients With Non-alcoholic Fatty Liver Disease

**DOI:** 10.7759/cureus.21110

**Published:** 2022-01-11

**Authors:** Bandana Kumari, Sadhana Sharma, Ramesh Kumar, Satish Dipankar, Bijaya N Naik, Ayan Banerjee, Sushil Kumar

**Affiliations:** 1 Biochemistry, All India Institute of Medical Sciences, Patna, Patna, IND; 2 Gastroenterology, All India Institute of Medical Sciences, Patna, Patna, IND; 3 Physiology, All India Institute of Medical Sciences, Mangalagiri, Mangalagiri, IND; 4 Community and Family Medicine, All India Institute of Medical Sciences, Patna, Patna, IND

**Keywords:** platelet distribution width, lipid ratios, non-alcoholic steatohepatitis, fibroscan, end stage liver disease, non-alcoholic fatty liver disease

## Abstract

Introduction

The clinical course of non-alcoholic fatty liver disease (NAFLD) in its long term may follow a benign course or have an adverse outcome leading to hepatocellular carcinoma (HCC) or end-stage liver disease requiring liver transplantation. Such patients represent only a small proportion of all NAFLD cases, making case finding a real challenge.

Aims

This study was planned to test the efficacy of simple laboratory parameters for their ability to screen advanced cases of NAFLD who need early attention to extricate them from the cumbersome outcome.

Material and method

The study protocol enrolled 129 diagnosed cases of NAFLD. Patients were categorized as group I with mild/moderate fibrosis (MF) comprising of F0 to F2 and group II with advanced fibrosis (AF) comprising of F3 and F4 based on Fibroscan kPa (kilopascal) score.

Results

Group I consisted of 96 MF patients, while group II included 33 AF patients. Mean values of alanine transaminase (ALT), aspartate transaminase (AST), alkaline phosphatase (ALP), triglyceride (TG), triglyceride/high-density lipoprotein (TG/HDL) ratio, total cholesterol/high-density lipoprotein (TC/HDL) ratio, and platelet distribution width (PDW) were significantly higher in patients with AF (group II), while platelet count (PC) was significantly lower in group II. The area under the receiver operative characteristic (AUROC) curve was highest for PDW [0.730 (0.644-0.815)] and TG/HDL ratio [0.719 (0.612-0.827)]. TG/HDL ratio at a cut-off of >2.4 had a sensitivity and specificity of 84.85% and 34.38%, respectively, and PDW at a cut-off of >16.40 had a sensitivity and specificity of 84.85% and 54.17%, respectively.

Conclusion

Decent sensitivity at particular cut-offs for TG/HDL ratio and PDW makes them suitable to be applied for screening advanced cases of NAFLD that require early ministration and medication to block its further progression to its intricate form.

## Introduction

Non-alcoholic fatty liver disease (NAFLD) is the most common form of chronic liver disease and can range from simple steatosis with a fortunate prognosis to a necro-inflammatory form, nonalcoholic steatohepatitis (NASH), in which cirrhosis results from accumulating fibrosis and can further progress to its arduous form, i.e., hepatocellular carcinoma (HCC) [1]. It has been estimated that 40.76% of NAFLD cases progress to fibrosis [2]. The incidence of HCC in patients with NAFLD is only 0.2% after eight years, but patients with advanced fibrosis (AF) have a 25-fold increased risk. The cumulative incidence of HCC in NASH and cirrhosis is 2.4% and 12.8% over a period of 3.2-7.2 years [1]. As the natural course of NAFLD to develop into its advanced form takes years, it requires continuous monitoring at regular intervals over a long period. A clinician is thus required to have a close follow-up of patients with NAFLD to capture the progression of early NAFLD to its advanced stage and to oversee potential advanced NAFLD in order to block its advancement to its complex form by early intervention.

Liver biopsy, although it is the gold standard test for evaluating fibrosis in patients with NAFLD, is invasive and is characterized by several limitations/risks; continuous monitoring of NAFLD progress and response to its treatment by repeated biopsy is unrealistic [3]. Transient elastography (Fibroscan), although a relatively decent approach, has very limited applicability, possibly due to its high cost and requirements for experienced personnel that limit its widespread use for follow-up of NAFLD cases in the general population in developing countries like India [4].

Researchers have previously reported the role of platelets in the prevention of hepatic fibrosis. Hepatic stellate cells (HSCs), activated myofibroblasts, and immune cells are involved in the pathogenesis of hepatic fibrosis. Platelets inhibit the activation of HSCs as well as the production of type I collagen in HSCs. Furthermore, platelet-derived growth factor inhibits the expression of type I collagen genes in HSCs and the expression of the transforming growth factor β (TGF-β) gene [5]. A decreased platelet count has been observed in patients with severe fibrosis due to reduced production of thrombopoietin by damaged liver cells and increased splenic sequestration and destruction of platelets. Because of the significant failure of platelets and increased interleukin-6 (IL-6) levels induced by inflammation, the platelet life cycle is shorter in patients with NAFLD [6]. The latter stimulates platelet production by bone marrow and promotes the release of larger, reticulated platelets into the bloodstream. This increased release of platelets causes an increase in the mean platelet volume and platelet distribution width (PDW) [7]. PDW obtained as a part of a simple automatic complete blood count may therefore provide laboratory measures for evaluating the progression of liver fibrosis.

The clinical burden of NAFLD is not only limited to the liver but can also affect the cardiovascular system of the patient [8]. Experimental evidence suggests that NAFLD, especially in its more severe form, exacerbates systemic and hepatic insulin resistance, causes atherogenic dyslipidemia, and releases proinflammatory, procoagulant, and pro-fibrogenic mediators that play an important role in the pathophysiology of cardiovascular abnormalities. Patients with NAFLD could thus benefit from more intensive surveillance and early treatment interventions to decrease the risk of cardiovascular complications. In addition, lipid ratios have a greater predictive value than isolated parameters of the lipid profile when used independently [9]. Structured lipid ratios can serve as markers for screening NAFLD progression to severe fibrosis and elucidate the correlation of NASH with cardiovascular risk prediction.

Currently, there is no specific treatment for NAFLD [10]. After substantial research toward this goal, a promising phase III trial in 2019 generated a prospect for the development of a new drug treatment that would prevent the progression of NAFLD to cirrhosis and other complications [11]. While expecting the approval of this treatment for NAFLD, the current strategy is that pharmacotherapy should be used for patients with NASH or with significant fibrosis in order to have a desirable impact on the global burden of NAFLD [12,13]. Nevertheless, as these patients represent only a small percentage of the total NAFLD cases, their identification in clinical practice is a real challenge. In addition, there are no well-characterized symptoms of NAFLD, and awareness of this disease in the general population is very limited [10].

Several prognostic scores based on serological markers have also been previously assessed and have been shown to be associated with liver severity, although none has been evaluated in depth yet [14].

The basic aim of the current study was to investigate the role of simple parameters like lipid ratios, such as triglyceride to high-density lipoprotein (TG/HDL) ratio, total cholesterol to high-density lipoprotein (TC/HDL) ratio, low-density lipoprotein to high-density lipoprotein (LDL/HDL) ratio, and PDW as possible predictors of advanced fibrosis in this increasingly prevalent, yet relatively unaddressed disease.

## Materials and methods

The study protocol enrolled 129 diagnosed cases of NAFLD based on radiological and biochemical findings that had also undergone a Fibroscan. Only adult subjects (age >18 years) with all the required data readily available were included in the study for analysis. This was a retrospective diagnostic accuracy study conducted at the All India Institute of Medical Sciences in Patna, Bihar, India after obtaining institutional review board approval. Informed consent was waived off due to the retrospective nature of the study.

Patients with an associated history of chronic liver disease, advanced liver disease, hepatic congestion, cardiac failure, and taking hepatotoxic drugs were excluded from the study.

Demographic data, including age and sex, were obtained for each subject. All relevant laboratory test results for the study were obtained from the hospital’s information system. Fibroscan test reports were obtained from the gastroenterology department. Table 1 presents the reference range of various laboratory tests included in the current study.

**Table 1:** Reference range of various laboratory tests TC: total cholesterol, TG: triglyceride, HDL: high-density lipoprotein, LDL: low-density lipoprotein, PC: platelet count, PDW: platelet distribution width, ALT: alanine transaminase, AST: aspartate transaminase, ALP: alkaline phosphatase

Laboratory parameters	Reference range
TC	<200 mg/dl
TG	<150 mg/dl
HDL	40–60 mg/dl
LDL	100–129 mg/dl
TC/HDL ratio	<4.5
LDL/HDL ratio	<3
TG/HDL ratio	<3
PC	150–450 × 10^3^/mL
PDW	10–17.9%
ALT	10–28 U/L
AST	0–31 U/L
ALP	30–90 U/L

Tests for AST, ALT, ALP, and lipid profile were performed on an auto-analyzer, the AU5800 Series Clinical Chemistry Analyzer, while PC and PDW were performed by the hydrodynamic focusing method.

Liver biopsy, although considered the gold diagnostic standard, is characterized by various limitations such as sampling error, high cost, high bleeding risk, and pain [15]. Transient elastography has emerged as a valuable non-invasive imaging modality to grade liver fibrosis with high sensitivity and specificity [16]. The efficacy of Fibroscan in assessing liver stiffness has transcended the gold standard of liver biopsy [3] as this technique explores approximately 100 times larger liver volume compared to the biopsy specimen, providing a more reproducible perspective [17]. For the purposes of this study, thus, Fibroscan has been used to grade liver fibrosis instead of liver biopsy.

Patients were subjected to Fibroscan on FibroScan 502 TOUCH, manufactured by Echosens, Paris, France. The reports were validated by two experienced clinicians. Fibrosis was measured in kPa (kilopascals) and the results were interpreted as follows [18]: F0 = 1-6 kPa, F1 = 6.1-7 kPa, F2 = 7-9 kPa, F3 = 9.1-10.3 kPa, and F4 = >10.4 kPa.

Patients were categorized into two groups based on kPa values [18]. Group I with mild to moderate fibrosis (MF) comprises F0 to F2. Group II with AF comprises F3 and F4. Levels of serum TC, TG, LDL, HDL, AST, ALT, ALP, PC, and PDW were recorded for all patients. Differences in these parameters were assessed in two groups with MF (group I) and those with AF (group II).

Statistical analysis

All statistical analyses were performed using Stata version 13 software (StataCorp LLC, Texas, USA). The patients' information was entered into Microsoft Excel (Microsoft® Corp., Redmond, WA) sheets for analysis. The categorical variables are presented as percentages. The Chi-square test was used to test an association between two categorical variables. The continuous data were assessed for normal distribution using the one-sample Shapiro Wilk test. Furthermore, the continuous data were presented as mean ± SD (standard deviation). The receiver operating characteristic (ROC) curve was plotted to obtain the area under the curve, the cut-off score, and the sensitivity of the cut-off score. A P-value of 0.05 or less was considered statistically significant.

## Results

For this study, records from 129 patients were retrieved and analyzed. Group I consisted of 96 mild to moderate patients (74%), while group II with AF included 33 patients (26%), as shown in Table 2.

**Table 2:** Grading of liver fibrosis according to kPa score

Grading according to kPa score	Frequency (n=129)	Percent
Group I		
F0 (0–5.9)	58	44.9
F1 (6–6.9)	21	16.3
F2 (7–9)	17	13.2
Total	96	74.4
Group II		
F3 (9.1–10.3)	7	5.4
F4 (> 10.4)	26	20.2
Total	33	25.6

Demographic details of the patients are presented in Table 3. Patients with AF showed a significantly higher mean age compared to patients with MF; 48.18 ± 14.04 vs. 37.39 ± 15.18, respectively (p-value = 0.0005). The majority of the recorded patients (76%) were males.

**Table 3:** Demographic details of NAFLD patients grouped on the basis of severity *Significant P-value

	Overall	Group I	Group II
Frequency, n (%)	129 (100)	96 (74.42)	33 (25.58)
Age, mean (SD)	40.15 (15.62)	37.39 (15.18)*	48.18 (14.04)*
Male, n (%)	98 (76)	72 (73)	26 (27)
Female, n (%)	31 (24)	24 (77)	7 (23)

Table 4 presents the laboratory parameters of patients in two groups. No significant differences between the two groups were noticed for TC, HDL, LDL, and LDL/HDL ratio. Nevertheless, mean value was significantly higher for ALT (56.30 ± 61.08 vs. 88.52 ± 96.56, p-value=0.02); AST (43.77 ± 50.89 vs. 89.92 ± 132.94, p-value=0.005); ALP (118.58±104.04 vs. 181.09 ± 210.96, p-value=0.02); TG (138.47±62.75 vs. 171.10±100.33, p-value=0.03); TG/HDL (2.94 ± 0.97 vs. 4.11 ± 1.5, p-value<0.0001); TC/HDL(4.0 ± 1.26 vs. 4.61 ± 1.84, p-value= 0.03); and PDW (15.64 ± 2.8 vs. 18.10 ± 1.80, p-value < 0.0001) among patients with AF (group II) compared to patients with MF (group I). The mean value was significantly lower for PC (216.01 ± 85.57 vs. 173.78 ± 85.71, p-value=0.01) among patients with AF compared to patients with MF. The mean kPa score of patients with AF and MF was 25.36 ± 21.93 and 5.48 ± 1.52, respectively.

**Table 4:** Laboratory details of NAFLD patients grouped on basis of severity *Significant P-value TC: total cholesterol, TG: triglyceride, HDL: high-density lipoprotein, LDL: low-density lipoprotein, PC: platelet count, PDW: platelet distribution width, ALT: alanine transaminase, AST: aspartate transaminase, ALP: alkaline phosphatase, NAFLD: non-alcoholic fatty liver disease

Laboratory parameters (unit)	Group I	Group II	P-value
Urea (mg/dl)	24.61 ± 14.18	30.30 ± 22.47	0.09
Creatinine (mg/dl)	0.85 ± 0.44	0.91 ± 0.44	0.49
ALT (U/L)	56.30 ± 61.08	88.52 ± 96.56	0.02*
AST (U/L)	43.77 ± 50.89	89.92 ± 132.94	0.005*
ALP (mg/dl)	118.58 ± 104.04	181.09 ± 210.96	0.02*
TC (mg/dl)	172.45 ± 56.29	188.11 ± 62.34	0.18
LDL (mg/dl)	98.12 ± 31.67	101.31 ± 42.20	0.64
HDL (mg/dl)	45. 08 ± 11.65	44.99 ± 17.15	0.97
TG (mg/dl)	138.47 ± 62.75	171.10 ± 100.33	0.03*
TG/HDL ratio	2.94 ± 0.97	4.11 ± 1.5	<0.0001*
TC/HDL ratio	4.0 ± 1.26	4.61 ± 1.84	0.03*
LDL/HDL ratio	2.29 ± 0.88	2.54 ± 1.17	0.19
PC (× 10^3^/mL)	216.01 ± 85.57	173.78 ± 85.71	0.01*
PDW (%)	15.64 ± 2.8	18.10 ± 1.80	<0.0001*
kPa score	5.48 ± 1.52	25.36 ± 21.93	<0.0001*

Table 5 presents the area under the receiver operator characteristic (AUROC) curve for different laboratory parameters. PDW showed the highest area under the curve [0.730 (0.644-0.815)] and TG/HDL ratio [0.719 (0.612-0.827)]. With respect to diagnostic characteristics, the TG/HDL ratio at a cut-off higher than 2.4 showed sensitivity and specificity of 84.85% and 34.38%, respectively, while PDW at a cut-off higher than 16.40 had sensitivity and specificity of 84.85% and 54.17%, respectively.

**Table 5:** Area under ROC curve for different laboratory parameters and diagnostic characteristics of TG/HDL ratio and PDW TC: total cholesterol, TG: triglyceride, HDL: high-density lipoprotein, LDL: low-density lipoprotein, PC: platelet count, PDW: platelet distribution width, ALT: alanine transaminase, AST: aspartate transaminase, ALP: alkaline phosphatase, AUROC: area under the receiver operative characteristic, CI: confidence interval

Laboratory parameters (unit)	AUROC curve	Asymptotic 95% CI	
		Lower boundary	Upper boundary
ALT (U/L)	0.580	0.461	0.700
AST (U/L)	0.630	0.506	0.753
ALP (U/L)	0.609	0.498	0.719
TC (mg/dl)	0.600	0.481	0.719
TG (mg/dl)	0.560	0.435	0.685
HDL (ml/dl)	0.494	0.375	0.614
LDL (mg/dl)	0.494	0.372	0.615
TC/HDL ratio	0.599	0.483	0.715
TG/HDL ratio	0.719	0.612	0.827
LDL/HDL ratio	0.560	0.434	0.687
PDW	0.730	0.644	0.815
Diagnostic characteristics of TG/HDL ratio and PDW	TG/HDL ratio	PDW	
Cut-off	>2.4	>16.40	
Sensitivity	84.85%	84.85%	
Specificity	34.38%	54.17%	
AUROC (CI)	0.719 (0.61–0.82)	0.73 (0.64–0.81)	

**Figure 1:**
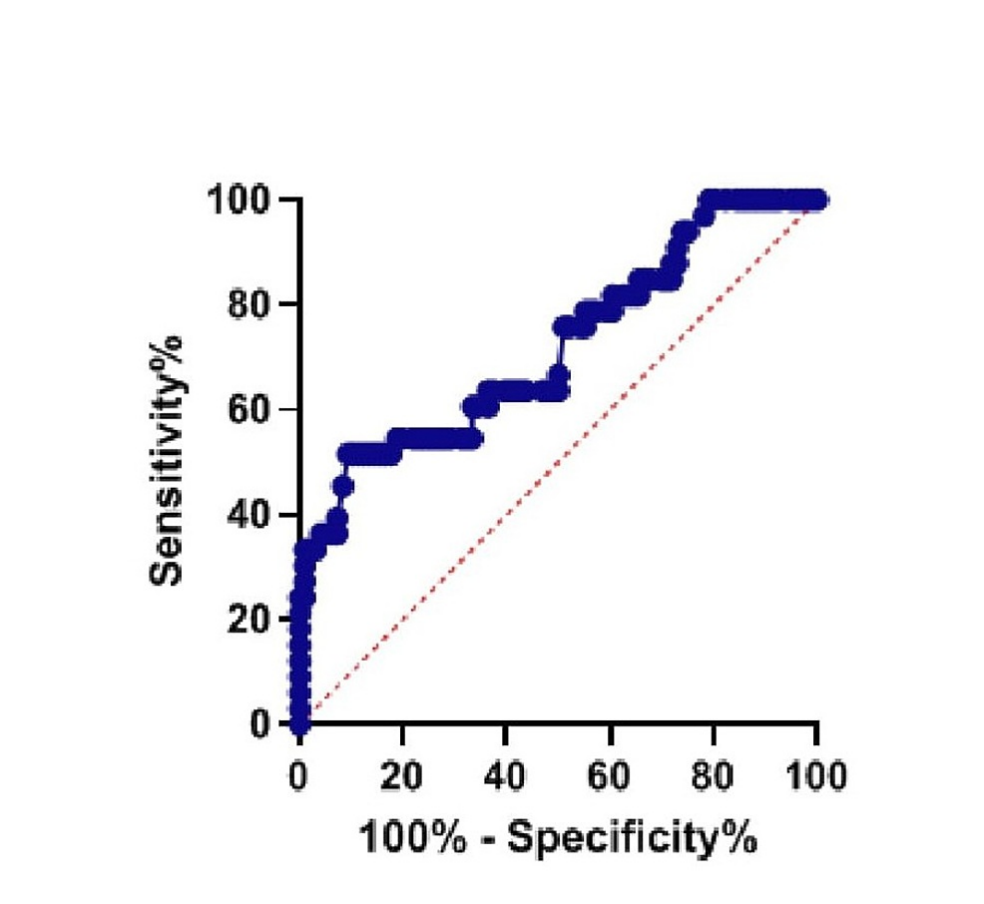
Represents the plotted ROC curve for TG/HDL ratio ROC: receiver operating characteristic, TG: triglyceride, HDL: high-density lipoprotein.

**Figure 2:**
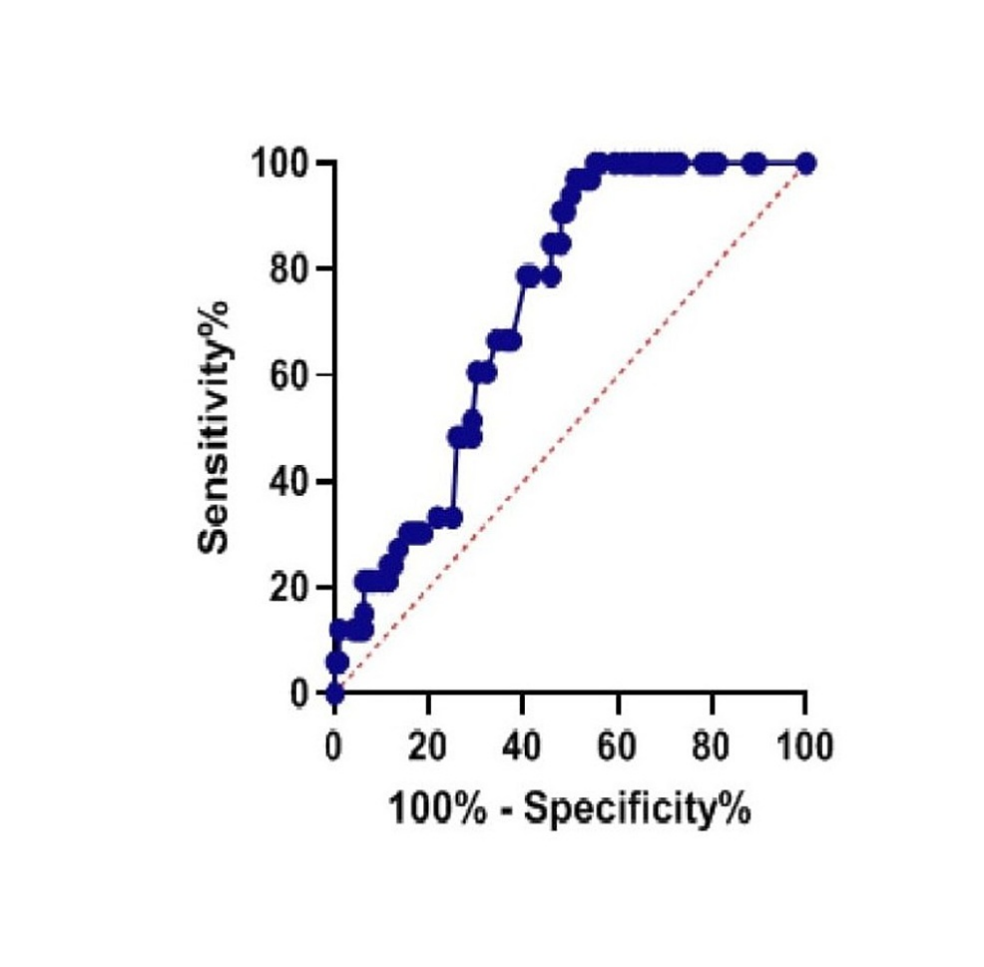
Represents the plotted ROC curve for PDW ROC: receiver operative characteristic, PDW: platelet distribution width

Figures 1-2 represent the plotted ROC curves for TG/HDL ratio and PDW, respectively.

## Discussion

In the current study, advanced stage fibrosis was linked to advanced age, something that is consistent with previous studies. This could be attributed to the increased affiliation of risk factors like hypertension, diabetes mellitus type II, and/or obesity with age [19]. The "free radical theory of aging" supports that reactive oxygen species generated in aged people cannot be eradicated, something that leads to increased oxidative stress levels in these people and to subsequent modification in cell growth and tumor suppressor genes [20]. Oxidative stress has been identified by researchers as one of the main culprits for the development of NASH, in addition to genetic factors and the gut microbiome. Patatin-like phospholipase domain-containing protein 3 (PNPLA3) is a genetic variant that has been found to be associated with steatosis, inflammation, cirrhosis, and HCC [21]. Moreover, different products released by bacteria in humans’ guts activate liver inflammation [22].

According to the results of the current study, NAFLD is more common in males than females, as female hormones appear to offer protection against NAFLD. Premenopausal women and women on hormone replacement therapy are less likely to develop NAFLD [23]. The performance of tests included in the lipid profile, lipid ratios, and platelet distribution among the two groups of patients was evaluated, which are simple biochemical tests regularly performed.

A statistical difference was observed in the values of PC and PDW in the two groups, as PC was significantly lower in group II and PDW was significantly higher in group II with a cut-off of >16.4 for the presence of severe fibrosis and sensitivity, specificity, and AUROC curve were 84.85%, 54.17%, and 0.73, respectively. A similar result was found in a study conducted by Milovanovic Alempijevic et al., in which PDW with a cut-off of 16.18 for the presence of severe steatosis had a sensitivity, specificity, and AUROC of 88.1%, 32.6%, and 0.688, respectively [14].

In our study, the levels of TG, TG/HDL ratio, and TC/HDL ratio were significantly increased among group II patients with AF. This finding suggests that dyslipidemia, as well as structured lipid ratios, could be utilized as markers of screening NAFLD progression to its severe form and confirms the association of cardiovascular risk with the advanced form of NAFLD.

AUROC had the highest value for TG/HDL ratio (0.719) among other lipid ratios, and with a cut-off higher than 2.4, it presented significant sensitivity (84.85%) but average specificity (34.38%) in the prediction of AF in patients with NAFLD. When using lower than these cut-off values, patients with mild/moderate NAFLD could be excluded.

In a study performed by Hegazy et al., with a cut-off of greater than 2.3, sensitivity, specificity, and AUROC were found to be at 91.7%, 64.2%, and 0.779, respectively [9]. The estimated cut-off for the TG/HDL ratio according to our studied population was higher than 2.4, similar to the study conducted by Hegazy et al., where the cut-off was higher than 2.3, significantly lower than the conventional cut-off for the TG/HDL ratio.

According to the study performed by Wakabayashi and Daimon, the conventional cut-off values of the TG/HDL ratio for men and women are 3.75 and 3.00, respectively, while the optimal cut-off for multiple cardiovascular risk factors in men and women is 2.967 and 2.237, respectively [24].

The newly detected cut-off values in the current study could be applied to screen every NAFLD case for its severity and association of cardiovascular co-morbidity. As NAFLD has an independent role as a cardiovascular risk enhancer [8], early detection and management might enable the prediction of cardiovascular disease morbidity occurrence.

A key limitation of this study is the relatively small sample size, while cut-off values require further large-scale validation in order to be perfectly legitimate. Lipid ratios and PDW can be helpful as markers of fibrosis progression only when these tests are repeatedly recommended to the patients on follow-up and not in a single setting. An increment in their values on follow-up will suggest progression of fibrosis.

## Conclusions

Decent sensitivity at particular cut-offs for the TG/HDL ratio and PDW makes them suitable to be applied for screening advanced cases of NAFLD that require early ministration and medication to block its further progression to its intricate form. As the TG/HDL ratio is linked to the advanced state of NAFLD cardiovascular risk, it could be used as a marker to pinpoint patients at menace for the timely detection and management of the disease in order to avoid the advancement of cardiovascular complications to an untreatable form.
